# Ontological insecurity in the post-covid-19 fallout: using existentialism as a method to develop a psychosocial understanding to a mental health crisis

**DOI:** 10.1007/s11019-023-10157-9

**Published:** 2023-06-30

**Authors:** Matthew Bretton Oakes

**Affiliations:** Department of Health and Applied Social Sciences, School of Social Sciences, Liverpool Hope University, Liverpool, UK

**Keywords:** Existentialism, Ontological insecurity, Covid-19, Mental health, Biopsychosocial, Psychosocial.

## Abstract

In the wake of the Covid-19 pandemic we are witnessing a significant rise in mental illness diagnosis and corresponding anti-depressant prescription uptake. The drug response to this situation is unsurprising and reinforces the dominant role (neuro)biology continues to undertake within modern psychiatry. In contrast to this biologically informed, medicalised approach, the World Health Organisation (WHO) issued a statement stressing the causal role of psychological and social factors.

Using the concept of ontological insecurity, contextualised within the WHO guidance, the interrelation of psychological and social factors is illuminated, and a psychosocial framework is produced as a means of understanding the mental health consequence of the post-Covid-19 fallout.

The psychosocial framework generated provides a rationale to revise and reprioritise how we engage with the biopsychosocial model that is intended to underpin modern psychiatry. This framework establishes a connection between psychological and social theory which are too often addressed as disparate terrains within mental health services and policy creation.

## Covid-19 and a mental health crisis

Before Covid-19 mental health was already described by Whitaker (2011) as the ‘epidemic’ of gravest concern facing the Western world. Supporting the accuracy of this highly sensationalised position, the House of Commons (2021, p. 6) stated that ‘prevalence [of common mental health disorders] has risen by around one-fifth in both men and women’ between 1993 and 2014. Further research between 1993 and 2017 evidenced a significant rise in the prescription of antidepressant medication^1^, from 1.9 to 10.1% (Bastiampillai et al., 2019).

During the Covid-19 pandemic^2^ several studies warned that this mental health situation was only likely to get worse (see Xiong et al., 2020; Sample, 2020), and the research since validates that concern. The UK Office of National Statistics reported a significant increase in adults experiencing common mental health disorders (CMD) in early 2021, a pre-pandemic base rate of 10% rising to 21% (ONS, 2021a). The NHS also reported that between March 2020 and June 2021 ‘1.2 million more antidepressant prescription items were issued than expected based on historical trends’ (NHSBSA, 2021).

Growing diagnosis rates and psychopharmacological prescriptions (anti-depressants etc.,) are different means of telling the same story; our mental health is deteriorating and even more so as a result of the Covid-19 pandemic. Furthermore, the continuing bond of diagnosis and psychopharmacology in the wake of this pandemic, informs us that the resultant emotional turmoil surfacing is being treated in the same biological model.

The application of purely biological models has done great harm to psychiatry, downgrading the importance of psychosocial factors. (Bracken et al., 2012, cited in Paris, 2015, p. 61)

Modern psychiatry has increasingly relied on the biological model. This is both from a perceived epistemic authority; the cause of mental illness believed to occur due to chemical imbalances within the brain, and through logistical necessity; drug treatment being the only viable means of responding to the increasing demand of mental illness diagnosis within contemporary culture. This dominance however, justified through necessity and/or perceived authority, is increasingly questioned (Rose, 2019; Davies, 2013; Moncrieff, 2008).

The COVID-19 pandemic reveals existing weaknesses in the mental health system, but also presents opportunities for reform.^3^ (Magoon et al., 2020)

This mental health crisis presents an opportunity to reassess the application of the biopsychosocial model within modern psychiatry, to reassess the default authority given to the biological model and emphasise and elevate the interplay of psychological and social factors. With the application of existential theory and the concept of ontological insecurity, a reanalysis of this current crisis can be drawn together into a psychosocial framework, offering new insight into how we approach its understanding.

## Ontological (in)security, mental illness and society

Ontological security is security not of the body but of the self, the subjective sense of who one is. (Mitzen, 2006, p. 344)

Ontological (in)security^4^ is not an obvious framework to consider the current mental health crisis, and this oversight is primarily because of the all-consuming narrative held by the biomedical model. However, as will be shown, not only can the current situation be described as destabilising our subjective sense of belonging to this world, the consequences are also causing significant mental health issues.

The concept of ontological (in)security was created by R.D. Laing, who stated that normal existence possessed a sense of ontological security^5^, and this meant that a ‘person will encounter all the hazards of life …from a centrally firm centre of his own and other people’s reality and identity’ (Laing, 1960, p. 39), summarised neatly in the quote above as *security in the subjective sense of who one is.* However, it was ontological *in*security and by default, mental illness, he focussed upon.

[For] the ontologically insecure person … the ordinary circumstances of living threaten his low threshold of security. (Laing, 1960, p. 42)

Caused by a perceived or actual threat (to *the subjective sense of who one is*), Laing defined ontological insecurity as a loss of subjective totality. This was illustrated most profoundly in the extreme of separating one’s inner world experience from outer world experience. Laing stated that this divided or split self, ontological insecurity, due to our biomedical framing was (mis)interpreted as mental illness (schizophrenia). Only through accurate understanding, can accurate, helpful, treatment be achieved.

‘Ontological insecurity’… is said to lie at the heart of serious mental illnesses. (Sedgwick, 1982, p. 15)

Laing’s concept, ontological (in)security, was ‘not a direct application of any established existential philosophy’ (Laing, 1960, p. 9), but his unorthodox theory provides a springboard to engage existentialism more thoroughly in the following section, enabling a more accurate analysis and thus understanding of our current predicament.

Before delving into the existential DNA of ontological (in)security, the ability to extend beyond Laing’s individualised framework needs to be considered. The collective application of ontological (in)security owes to Anthony Giddens, specifically his seminal text, *Modernity and Self-Identity.*^6^ Stating that ‘for the first time in human history, ‘self’ and ‘society’ are interrelated in a global milieu’ (1991, p. 32), he outlined that global conditions shaped our ontological (in)security on a collective scale. Contrasting with Laing, he asserted that the normal conditions of modern society were no longer a producer of ontological security, and our collective resilience to navigate the anxieties associated with everyday existence – our resistance to madness, amongst other subjective foibles – had become compromised. Whilst madness is an obvious inroad to mental illness, Giddens’ theory provided little more than a nod toward this connection or development for mental illness. Giddens’ theory however demonstrated the potential for ontological (in)security to understand society collectively.

As previously discussed, mental illness diagnosis has increased significantly post-March 2020. Of interest, this is also mirrored with research into ontological insecurity. Several articles have been published in the aftermath of the Covid-19 pandemic, and whilst different sub-focuses are offered, they agree that ontological insecurity has also increased in this timeframe (see Wright et al., 2021; Agius et al., 2020; Leary et al., 2022; etc.). A correlation is no guarantee of a causal relationship, but the potential of this link strengthens with a refocussed application of ontological (in)security.

To justify the value of ontological (in)security as a means of understanding the current post-Covid-19 mental health crisis, an argument needs to be built that this pandemic has caused a perceived or actual threat to the *subjective sense of who one is*. This is the proposition of this article and arguing this claim requires a closer look at the human condition within ontological (in)security.

## The DNA of ontological (in)security

[1] man is free, in the sense of being compelled to choose between infinite possibilities… [2] he experiences his freedom in anguish… [3] the unpleasantness of this state motivates man to hide the fact of his freedom from himself by absorption in the conventional practices of everyday life (‘inauthenticity’). (Collier, 1977, p. 17)

Collier’s meta-summary of the human condition is helpful in its simplicity. This framework identifies three base experiences that can unite the philosophical canon of Heidegger, Kierkegaard, and Sartre; a trilogy of influences that played an instrumental role in the conceptual development of ontological insecurity. Each of these elements play a major role within the experience of Covid-19, and by analysing each, a deeper basis by which to consider the factors at play within the fallout can be outlined.

[1] Heidegger (1927) referred to our potential free self as ‘*Being’* (denoted with a capital ‘B’). This is our most raw, pure, and essential self: the freedom to exist without constraint, restriction, or the perceived necessity to adhere to social norms. Awareness for an internal freedom to live without restraint – *Being*, by default, and conversely, increases awareness for the imminence and unavoidability of our mortality; to live is to die. To use a more Sartrean term; an inseparability of *Being* and nonbeing (Sartre 1957).

[2] Directly and intimately connected to this first element, the unknown and unknowable potential of *Being* (and thus nonbeing), existential thought recognises angst as a constant undercurrent of existence.

This is where the biomedical model and existentialism meet, and importantly diverge. Focussing on their shared ground firstly, each acknowledges a proximity for anxiety with fear:

[Fear] refers to something definite; where anxiety is freedom’s actuality as the possibility of possibility. (Kierkegaard, 1844, p. 51)

Fear is the emotional response to real or perceived imminent threat, whereas anxiety is anticipation of future threat. (APA, 2013, p. 189)

Their terminology may be different, but the essence underpinning each is the same. Fear is an emotional response to something definite, relating this back to Mitzen’s earlier definition – a threat to bodily security. Anxiety is located in the unknown, the abyss of potential – the epitome of ontological insecurity. Both play a role in the Covid-19 fallout.

The medical model appreciates a certain level of fear/anxiety is a positive attribute, promoting human survival (fight/flight response, etc.). However, a diagnosis for an ‘anxiety disorder’^7^ is given when fear and/or anxiety detrimentally affect a person’s day-to-day functioning. The recommended treatment for this diagnosis is psychotherapy and/or psychopharmacology. Unfortunately, due to reasons discussed earlier, drugs are the inevitable outcome (Rose, 2019; Davies, 2013; Moncrief, 2008).

Existentially, angst takes focus and is a double-edged sword in ontological (in)security. Angst is always rippling beneath the surface, and its awakening into conscious experience is not necessarily a bad thing; it can be a catalyst for gaining truer knowledge of self, realising one’s potential, a pathway to enlightenment. However, nobody said that this was an easy path to take. Sartre (1957) illustrates this difficulty profoundly:

The goal of bad faith … is to put oneself out of reach; it is an escape. (Sartre, 1957, p. 89)

‘Bad faith’ is the act of lying to oneself. It is a vital component which Sartre uses to underpin the human condition. Bad faith justifies how we live at one and the same time with the awareness of potential freedom but, leading to our final point, willingly remain immersed within the estrangement of worldliness, all in the avoidance of existential angst.

[3] In avoidance of existential angst, like an ostrich hiding its head in the sand to avoid danger, *Being* seamlessly immerses itself ‘in the conventional practices of everyday life’. This for Heidegger (1927) is the definition of ‘being-in-the-world’; the realisation of Dasein.

Dasein […] has the inclination to be ensnared in the world in which it is and to interpret itself in terms of that world by its reflected light. (Heidegger, 1927, p. 24)

Dasein accepts an absolute immersion within worldliness *(authentic commonality)* at the cost of potential existential freedom *(individual authenticity)*. At this juncture, a rigidly existential deconstruction runs into difficulty; *individual inauthenticity* does not fully align with any state of security. However, Giddens and Laing remind us that ontological security is not dependent on individual authenticity; ontological security relates to ‘a fundamental sense of [subjective] safety in the world’ and ‘a sense of psychological well-being and [avoidance of] existential anxiety’ (Giddens, 1991, p. 37).

Ontological security, at its simplest, is the maintenance of a careful balance for each of the three elements of the human condition. Their relative harmony regulating existential angst within conscious experience and securing a *subjective sense of who one is*. The problem is, Covid-19 has threatened *the subjective sense of who one is*, and arguably the fallout of this, angst, is being absorbed within a deficit, biomedical model of mental illness evidenced in the increased diagnosis rates of CMDs. This threat and resultant outcome are vividly illustrated in a statement issued by the World Health Organisation.

## Fear and angst in the covid-19 fallout

The global scientific authority of the World Health Organisation (WHO) has long been appreciated within research and professional circles. In the wake of the Covid-19 pandemic this authority has become more visible and connected to the general population. Their issuing of a statement on mental health at this time is a demonstration of the concern felt toward this specific aspect of the crisis.

Fear, worry, and stress are normal responses to perceived or real threats, and at times when we are faced with uncertainty or the unknown. So it is normal and understandable that people are experiencing fear in the context of the COVID-19 pandemic.

Added to the fear of contracting the virus in a pandemic such as COVID-19 are the significant changes to our daily lives as our movements are restricted in support of efforts to contain and slow down the spread of the virus. Faced with new realities of working from home, temporary unemployment, home-schooling of children, and lack of physical contact with other family members, friends and colleagues, it is important that we look after our mental, as well as our physical, health. (WHO, 2020)

This was a message regarding mental health they wanted the world to hear, offering a clear and understandable explanation to make sense of the difficult emotions people were experiencing. Crucially, it stressed that these emotions were both logical and reasonable. Furthermore, with a notable absence for any consideration toward a biological rationale to the mental health fallout of this pandemic, it begs the question as to whether the biological model is the most accurate to understand this crisis and more so, psychopharmacology as the most accurate treatment response.

From the outset of this statement, Covid-19 is presented as an unforeseen and unknown threat. This was a viral illness of unknown origin surrounded by uncertainty as to what were its full effects, or who it affected most vehemently. Existing with uncertainty is the epitome of existential anxiety, this experience alone unsettling the *sense of who one is* and therefore ontological insecurity. However, a more significant, deeper-seated, existential destabilising of the human condition becomes apparent as we analyse the role of fear and anxiety within this pandemic.

Their statement directed focus not toward the virus itself but the effects resulting from living through this unprecedented situation, and two prominent areas of concern overlapped with the biopsychosocial framework that underpins modern psychiatry (Paris, 2015); an instinctual fear of illness and/or death – a psychological element (para 1); the implications and limitations which stem from the announcement of lockdowns, representing a change to our environmental conditions – a social element (para 2). Addressing each paragraph independently, before collectively, through the application of ontological (in)security a psychosocial framework is revealed, creating valuable insight into this situation.

### The inner world of fear and angst

The most tangible and known threat was that of the risk of death and this is exemplified in the tone and language of paragraph 1. Typified by daily news announcements informing the UK population of the 24-hour Covid-19 related death rate (a figure that reached 72,299 deaths in the year 2021 - ONS, 2021b), the fear of death, our own, and/or our loved ones, became omnipresent. Furthermore, death was mobilised by the government as justification and leverage to gain compliance with transmission reduction strategies – namely, ‘working from home, temporary unemployment, home-schooling of children, and lack of physical contact with other family members, friends and colleagues’ etc. (WHO, 2020).

The risk to life was definite: a fear compromising security of body. Fear and bodily security do not fall seamlessly within a definition of ontological (in)security, but a less obvious, and arguably more potent, existential threat starts to unravel as we question fear of mortality further. Our imminent death, a heightened awareness of nonbeing, as previously discussed, necessarily heightens awareness for *Being*. The possibility of *Being*, our potential free self, induces anxiety, or to borrow from Kierkegaard (2014, p. 51) ‘anxiety is freedom’s actuality’. The Covid-19 pandemic increased awareness of *Being* and nonbeing, and death entered our consciousness on a scale not seen since the second world war (Smyth, 2021).^8^

Framed as fear of death or an anxiety of *freedom’s actuality* (*Being*), independently and collectively, a primitive and innate instinctual drive residing within the inner world of a persons’ psyche is revealed through this experience, a base psychological function within the human condition.

### Social angst and the outer world

The viability for an application of ontological (in)security is further illustrated in the second paragraph, with the counterbalance of the human condition coming into focus: ‘Added to’ an increased mortality awareness, we find ourselves doubly compromised in the outer, social world, with a necessity for adaptation to ‘significant changes to our daily lives’ (WHO, 2020).

‘To contain and slow down the spread of the virus’ and reduce the death toll (WHO, 2020), on 26th March 2020, UK wide lockdown measures legally came into force (IoG, 2022). Society was effectively placed under house arrest. Movement outside of the home was restricted to essential purposes only; food shopping, one hour of exercise per day, no meeting with persons outside of the household, etc. Developments to these restrictions unfolded as the pandemic dynamics changed, but until December 2021, anything resembling a normal social life was a distant memory. From a lack of participation in leisure activities, to simply missing the intimacy and relatedness of a handshake, kiss, or hug with friends and family; living in lockdown exerted a great toll on our mental health (Catling et al., 2022; Waite et al., 2021; Pierce et al., 2020). It must also be recognised that this was a stratified toll, disproportionately affecting those along a social gradient (ONS, 2021a), especially affecting those with a pre-existing mental illness diagnosis (Neelam et al., 2021).

Social theory has long advocated the benefits of the environment on mental health, the two existing in positive correlation; the better our socioeconomic conditions, the better our mental health (see Rogers and Pilgrim 2003; Wilkinson and Pickett, 2018; Compton and Shim 2019). But it is not simply a case of the environment providing greater opportunities to engage with generic health behaviours that translate into positive mental health – as per research surrounding the social determinants of mental health (ibid.). The existential underpinning of ontological (in)security adds a more detailed and nuanced appreciation as to *why* the environment impacts upon our mental health; a suitably nurturing environment is needed to provide refuge from the base existential angst caused by the awareness of *Being* and nonbeing. Our social environment provides solace and refuge from our most innate psychological drive, our potential freedom (*Being*) and thus unavoidable death (nonbeing). Giddens (1991) explains this as ‘bracketing’, the role of our social environment is to ‘bracket’ out the big questions about human existence.

The comfort blanket of our social immersion, the necessary counterbalance to ‘bracket’ awareness of *Being* and nonbeing, was found wanting. The social disruption of the pandemic denied us the familiarity and security of an existential home and so our resilience to angst was, and continues to be, compromised. The increased awareness of mortality, *Being* and nonbeing, already destabilised the human condition, and when we ‘add to’ that equation a drastic change to our worldly existence, our entire existential stability was set in flux.

### A psychosocial perspective

A limitation could be suggested with an implied linearity to the structure of ontological (in)security and reflected in the ordering of the WHO statement; fear of freedom/death comes first, causing angst, therefore we immerse ourselves in the everyday. Whereas the wealth of research emphasises environmental changes, specifically the impact of lockdown related social restrictions, first and foremost (Catling et al., 2022; Waite et al., 2021; Pierce et al., 2020; Suleman et al., 2021).

The potential for an application of ontological (in)security lies in its ability to consider the entirety of human experiences and conceptualise the person as a totality. Rather than an ordered process, or furthermore, an atomised psychological *or* social approach, this existential approach reaffirms that both inner and outer worlds operate as mutually dependent, one cannot be separated from the other.

[A psychosocial approach] seeks to transcend the dualism of the individual and the social … without engaging in either psychological or sociological reductionism. (Roseneil, 2006, p. 847)

Covid-19 may have disrupted and destabilised a psychological (*Being* and nonbeing) and/or social aspect (‘absorption in the conventional practices of everyday life’), but it has desynchronised a precariously balanced psychosocial self, affirming its qualification as a psychosocial framework. Without disciplinary reductionism, disruption can destabilise from any direction within the totality. Appreciating the psychosocial credentials of ontological (in)security enables new insight into how we understand the fallout of this pandemic.

## Pre-existing ontological insecurity

As society adjusts to a new-normal of ‘living with the virus’ (Gov.uk, 2022), the shadow of the pandemic continues to loom large, and presuming ontological security was once found in the refuge of everyday life, it is now destabilised. Existential angst has entered our experience more intensely, and we are struggling to ‘encounter all the hazards of life’ – decoded into medicalised psychiatric discourse, we are witnessing an increased incidence of mental illness diagnosis.

We must consider the possibility that this pandemic is acting upon an existing ontologically insecure position, a position Giddens and many others would endorse (Wright et al., 2021; Agius et al., 2020; Leary et al., 2022). Even joining the dots outside of Giddens et al., with a year-on-year increasing incidence of mental illness diagnosis, based on data collated from 1993 onwards (Bastiampillai et al., 2019; ONS, 2021a; NHSBSA, 2021), an existential argument can be presented that the ‘ordinary circumstances of living’ are already challenging our ‘threshold of security’. Even if we approach the post-Covid-19 fallout from a position of possessing ontological security, the mental health epidemic suggests we were already precariously close to a tipping point.

If we build from a position that ontological insecurity being the norm, the necessity and capability to adapt and manage ‘significant changes to our daily lives’ with an already psychosocially destabilised human condition, it is little wonder that those with pre-existing mental illness diagnoses were most affected by Covid-19’s social impact (Neelam et al., 2021; Suleman et al., 2021; MIND, 2021). With resilience incapacitated, there was no existential reserves to buffer the additional social strain.

However, the argument of whether we collectively possess ontological security or not, is a moot point. This pandemic has been shown to possess all the ingredients to create its absence, compromising all elements of the human condition. This provides a different, and arguably more accurate, means of understanding the increased CMD diagnosis rates.

## Summary

This article has highlighted the value for an application of ontological (in)security in response to the post-Covid-19 mental health fallout. Deconstructing the human condition, the role of anxiety within ontological security comes into the foreground. This allows us to reframe the medicalised symptom of anxiety in the existential experience.

The deconstruction of the human condition through established philosophical theory reveals both a psychological and a social component to our mental health; factors that have been disrupted during this period.

Without any attempt to dismiss the biomedical model, this highly existential approach presents an argument to invest more authority into a psychosocial understanding of the emotional difficulties experienced in the wake of this pandemic.

Having looked at how we can locate and understand the causation / onset of ontological insecurity the next logical step is to consider action, moving from understanding to treatment. The hope is that more accurate understanding leads to more effective treatment and better help.

Suleman et al. (2021) published, *Unequal pandemic, fairer recovery: The COVID impact inquiry report.* Within a comprehensive analysis addressing wide ranging factors affecting all aspects of health, including the mental health impact of social restrictions, they highlight that investment and policy must improve ‘the conditions needed for sustaining a healthy population’ (p. 78).

Whilst this social based research provides a powerful foundation, ontological (in)security develops this potential further by drawing in a psychological element.

The outcome of Suleman et al.’s research culminates with the recommendation to improve our social environment, essentially providing a safe existential refuge from our inner struggle (the potential of *Being* and nonbeing). This is the critical requirement to maintain balance.

With a sufficiently nurturing environment, whilst angst remains embedded within existential experience, we can avoid a rupturing into conscious awareness.

Moving a step on from Suleman et al.’s recommendation, we can take Hannigan and Coffey’s (2011) statement that good policy creation requires stronger interagency partnerships. An existential approach has the ability to create a shared theoretical terrain across different agencies and a common language for psychosocial discussions to develop.

Modern psychiatry has rejected its long-standing psychosocial perspective and has adopted a narrow version of the medical model. (Paris, 2015, p. xii)

The compatibility for ontological (in)security to offer a valuable understanding of this crisis, justifies widening our scope beyond the medical and reengaging a more psychosocial perspective.

## Footnotes

1 Antidepressant medication being a multipurpose prescription used for several CMDs, including depression and generalised anxiety disorder (NHS, 2021) – the two disorders seeing the greatest diagnosis rise in the post-Covid-19 fallout.

2 The peak impact of Covid-19, March 2020 – December 2021 (IoG, 2022).

3 Magoon et al., (2020) remain committed to the epistemic authority of the existing psychiatric services.

4 The term ‘(in)security’ is adopted in this paper to refer to the full concept, encompassing both security and insecurity.

5 The prefix a ‘sense of ontological insecurity’ was introduced in *The Politics of Experience* (1967). This pre-fix helps provide continuity with later publications where he also stated it was ‘mad to be normal’ (see Oakes 2021); a sense of ontological was equally an immersion within a mad society. This prefix also addressed a critique that ‘Laing confused the ontic with the ontological’ (Deurzen, 1997, p. 219). Whether his theory would have aligned with Giddens in the years passing his publications is a conversation that can only be speculated upon.

6 Gidden’s work has stimulated a wealth of research, see *European Journal of International Relations*, for numerous examples.

7 Several different types of anxiety disorders are listed in the DSM-5; including generalized anxiety disorder, separation anxiety disorder, panic disorder, social anxiety disorder, specific phobias, agoraphobia, social anxiety disorder, etc.

8 Interest in existentialism surged in the immediate aftermath of the second world war (Lawlor 2010), supporting a proposition that this philosophy may provide a proven discourse to think through times of increased mortality awareness.
